# Depiction of Vaginal Microbiota in Women With High-Risk Human Papillomavirus Infection

**DOI:** 10.3389/fpubh.2020.587298

**Published:** 2021-01-08

**Authors:** Zhen-Tong Wei, Hong-Liang Chen, Chun-Feng Wang, Gui-Lian Yang, Shu-Mei Han, Song-Ling Zhang

**Affiliations:** ^1^Department of Oncologic Gynecology, The First Hospital of Jilin University, Changchun, China; ^2^College of Veterinary Medicine, Jilin Provincial Engineering Research Center of Animal Probiotics, Jilin Agricultural University, Changchun, China; ^3^Medical Department, The First Hospital of Jilin University, Changchun, China

**Keywords:** persistent infection, *16s* rRNA, bacterial vaginosis, vaginal microbiota, human papillomavirus

## Abstract

Persistent infection with the carcinogenic human papillomavirus (HPV) is a prerequisite for the progression of cervical lesions and cancer. A growing body of research has focused on the functional role of the vaginal microbiota in the persistence of HPV infection. Understanding the microbial composition and structure in women with high-risk (hr)-HPV infection may help reveal associations between the vaginal microbiota and HPV infection, and identify potential biomarkers. Our study investigated the vaginal microbial community in women with and without hr-HPV infection, by using *16s* rRNA gene sequencing. We found that microbial perturbations occurred in the early phase of hr-HPV infection. *Lactobacillus* and *Sporolactobacillus* were decreased, while bacteria related to bacterial vaginosis (BV), such as *Gardnerella, Prevotella, Dialister, Slackia, Actinomyces, Porphyromonas, Peptoniphilus, Anaerococcus, Peptostreptococcus, Streptococcus, Ureaplasma, Megasphaera*, and *Mycoplasma* were increased. Our results could offer insights into the correlations between hr-HPV and the vaginal microbiota in the early infection period, and provide indications that the predominance of some BV-associated bacteria during hr-HPV infection may increase the risk for cervical neoplasia.

## Introduction

Globally, cervical carcinoma is the fourth most common type of cancer among women, after breast cancer, colorectal cancer, and lung cancer (1). Persistent infection with the oncogenic human papillomavirus (HPV) is the main factor responsible for the progression of cervical lesions and cervical cancer (2, 3). HPV is a non-enveloped DNA virus that infects human epithelial tissues. In approximately 10% of women infected with HPV, the virus is not completely cleared from the body, leading to persistent high-risk HPV (hr-HPV) infection (4). Infection with an hr-HPV genotype is considered one of the most important causes of cervical cancer. For instance, HPV16 and HPV18, two hr-HPVs, are responsible for approximately 55 and 15% of all cases of cervical cancer, respectively. HPV16 infection is mainly related to the occurrence of cervical squamous cell carcinoma (5, 6). In addition, douching practices, sexually transmitted infections, vulvovaginitis, vulvitis, and bacterial vaginosis (BV) perturb the vaginal microenvironment and likely serve as cofactors for persistent hr-HPV infection (7, 8). Other factors correlated with HPV persistence include age, smoking, immunodeficiency, contraceptive use, and infection with *Chlamydia trachomatis* (9, 10).

Highly diverse microbial communities at specific sites in the human body play vital roles in physiology, immunity, nutrition, and development, and are generally considered a sign of health (11, 12). Of these microbial communities, the gut microbiota is the best characterized, and has been implicated in certain diseases as well as in carcinogenesis (13). The Human Microbiome Project investigated whether the vaginal microbiome was correlated with human health and disease (14). *Lactobacillus* spp. are commensal bacteria in the vaginal microbiome that can help defend against sexually transmitted infections and other pathogens by maintaining a hostile pH, producing species-specific metabolites and bacteriocins, adhering to mucous membranes, and disrupting biofilms (15, 16). Most previous studies on vaginal microbiota focused on analyzing the compositions of the vaginal microbiota in healthy women and women with BV (17, 18). Recently, however, it was found that the vaginal microbiota was correlated with HIV infection (19–21). The precise associations between the vaginal microbiota and HPV infection, and the key bacterial population changes relevant to the development of cervical carcinoma remain unclear.

The majority of studies on vaginal microbiota have relied on cultivation-dependent methods to assess microbial community composition (22). Recently, however, sequencing technologies have been used to investigate the human microbiome at various body sites, and these methods enable a more direct and rapid analysis of microbial taxa (23). In the present study, we used *16S* rRNA gene sequencing to comparatively analyze the vaginal microbiota in 30 HPV-negative women and 30 HPV-positive women with normal cytology in the northeast of China, in order to reveal potential biomarkers in the early phase of HPV infection.

## Methods

### Study Population

We recruited a total of 60 women who underwent routine gynecological examination in the Department of Oncologic Gynecology, the First Hospital of Jilin University, Changchun, China, between September and December 2018. The exclusion criteria included current pregnancy, menopause, sexual intercourse or vaginal lavage within 3 days, use of antibiotics or vaginal antimicrobials within 2 weeks, HIV infection, and autoimmune disorders. All subjects were interviewed to collect the following information: age, ethnicity, smoking habits, parity, and contraceptive use. In addition, all subjects underwent cervical ThinPrep cytology tests, regardless of HPV infection status, and were found to have normal cytology without cervical intraepithelial neoplasia (CIN). All participants provided written informed consent for the use and publication of their data prior to their enrollment into the study. The study protocol was approved by the ethics committee of the First Hospital of Jilin University, and the study was conducted in accordance with the principles of the Declaration of Helsinki.

### Sample Collection and HPV Detection

Three swab specimens were collected from each participant during a routine pelvic examination. The first specimen was collected from the cervix with a Cytobrush and used for cervical ThinPrep cytology testing (Hologic, Marlborough, MA, USA). The second specimen was also collected from the cervix and used for hr-HPV DNA detection via the Hybrid Capture II (HC2) test (Qiagen, Gaithersburg, MD, USA), according to the manufacturer's guidelines. The HC2 test is a chemiluminescence-based assay that involves nucleic acid hybridization and signal amplification. This test can qualitatively detect 13 types of hr-HPV DNA, namely, 16, 18, 31, 33, 35, 39, 45, 51, 52, 56, 58, 59, and 68. The third specimen was collected from the mid-vagina, and was immediately frozen to−80°C and subsequently used for bacterial DNA extraction and sequencing.

### *16S* rRNA Gene Sequencing

We extracted genomic DNA from a total of 60 cervical samples by using E.Z.N.A.® DNA extraction kits (Omega Bio-Tek, Norcross, GA, USA), according to the manufacturer's instructions. Next, we verified the quality of the extracted DNA by performing 1% agarose gel electrophoresis, and measured its concentration using NanoDrop spectrophotometry. The extracted DNA was then frozen to −20°C and stored until further analysis. We then performed high-throughput sequencing of the V3–V4 hypervariable regions of the bacterial *16S* rRNA gene on the Illumina MiSeq PE300 sequencing platform (Illumina Inc., San Diego, CA, USA). Amplification of the V3–V4 regions was performed with the following pair of universal primers, which contained a set of 8-nucleotide barcode sequences unique to each sample: forward, 338F (5′-ACTCCTACGGGAGGCAGCAG-3′); and reverse, 806R (5′-GACTACHVGGGTWTCTAAT-3′). The polymerase chain reaction (PCR) protocol was as follows: 5 min at 95°C, 25 cycles of 30 s at 95°C, 30 s at 55°C, and 30 s at 72°C, and final extension for 10 min at 72°C. PCR was performed in a 25-μL mixture containing 2.5 μL of 10 × Pyrobest buffer, 0.4 U Pyrobest DNA Polymerase (TaKaRa, Shiga, Japan), 2 μL of 2.5 mM dNTPs, 15 ng template DNA, and 1 μL of each primer (10 μM). All PCR assays were performed in triplicate. The resulting amplicons were subjected to 2% agarose gel electrophoresis, purified (AxyPrep DNA Gel Extraction Kit, Axygen Biosciences, Union City, CA, USA), and then quantified (QuantiFluor™ -ST, Promega, Madison, WI, USA), according to the manufacturers' instructions. Finally, the purified amplicons were pooled in an equimolar ratio and paired-end sequenced (2 × 300) on the Illumina MiSeq platform.

### Bioinformatics and Sequencing Data Analysis

We used QIIME (Quantitative Insights Into Microbial Ecology, *version* 1.2.1; http://qiime.org/) to extract high-quality sequences from the raw sequences obtained using MiSeq sequencing. Sequence selection was based on the length and quality of the sequences, tags, and primers. The set of unique sequences thus identified was categorized into operational taxonomic units (OTUs; threshold, 97% identity) by using UCLUST (*version* 1.2.22; https://drive5.com/usearch/manual/uclust_algo.html). Using Usearch (*version* 8.0.1623; https://www.drive5.com/usearch/), we identified and removed chimeric sequences. Taxonomic identification of the remaining *16S* rRNA gene sequences was performed using the Silva119 *16S* rRNA database and UCLUST software (confidence threshold, 90%).

We evaluated the Chao1 and observed species indices, which estimate microbial richness, and the Shannon index, which measures species biodiversity (24, 25). These three indices were used to calculate the alpha diversity of the samples. Principal component analysis of OTUs was performed using the R software (*version* 3.6.0, Revolutions; https://www.r-project.org/), and co-occurrence network analysis was performed using the R packages “igraph” and “psych.” To identify significant differences in microbial composition, we performed linear discriminant analysis effect size (LEfSe; http://huttenhower.sph.harvard.edu/galaxy/) with the thresholds *P* < 0.05 and effect size > 2. Clusters of Orthologous Groups (COG) categories were assigned by performing BLAST searches to identify the closest matching sequence in the Search Tool for the Retrieval of Interacting Genes database. The raw reads were deposited into Sequence Read Archive (NCBI accession number: SRP212297).

### Statistical Analysis

Experimental comparisons were performed using analysis of variance and the Tukey honestly significant difference test. The results are presented as mean and standard error of the mean. *P* < 0.05 was considered to indicate statistically significant differences.

## Results

### Participants' Characteristics

Of the 60 women, 30 tested negative for hr-HPV infection, while the remaining 30 tested positive for hr-HPV infection. The average ages of HPV-positive and HPV-negative women were 36.70 ± 6.30 and 34.13 ± 6.43 years, respectively. All cytological tests were normal, and no CIN lesions were detected in any of the women from either group. The HPV-positive and HPV-negative groups did not differ in terms of age, parity, ethnicity, cigarette smoking, or use of hormonal contraceptives. The demographic characteristics of each group are presented in Table 1.

**Table 1:** Demographic characteristics of the HPV-positive and HPV-negative groups.

**Characteristics**	**HPV Positive group (*n* = 30)**	**HPV negative group (*n* = 30)**
**Age (years)**		
20–29	4	9
30–39	13	13
40–49	13	8
**Ethnicity**		
Ethnic Han	30	28
Ethnic of Korean minority	0	2
**Smoking habits**		
Current smoker	1	1
Non-smoker	29	29
**Contraception**		
Nil	4	4
Condoms	15	19
IUD	10	5
Natural contraception	1	2
Hormonal contraception	0	0
**Parity number**		
0	7	7
1	17	19
2	6	4
3	0	0

*HPV, human papilloma virus; IUD, intrauterine device*.

### Vaginal Microbial Composition in HPV-Positive and HPV-Negative Women

**Figure 1:**
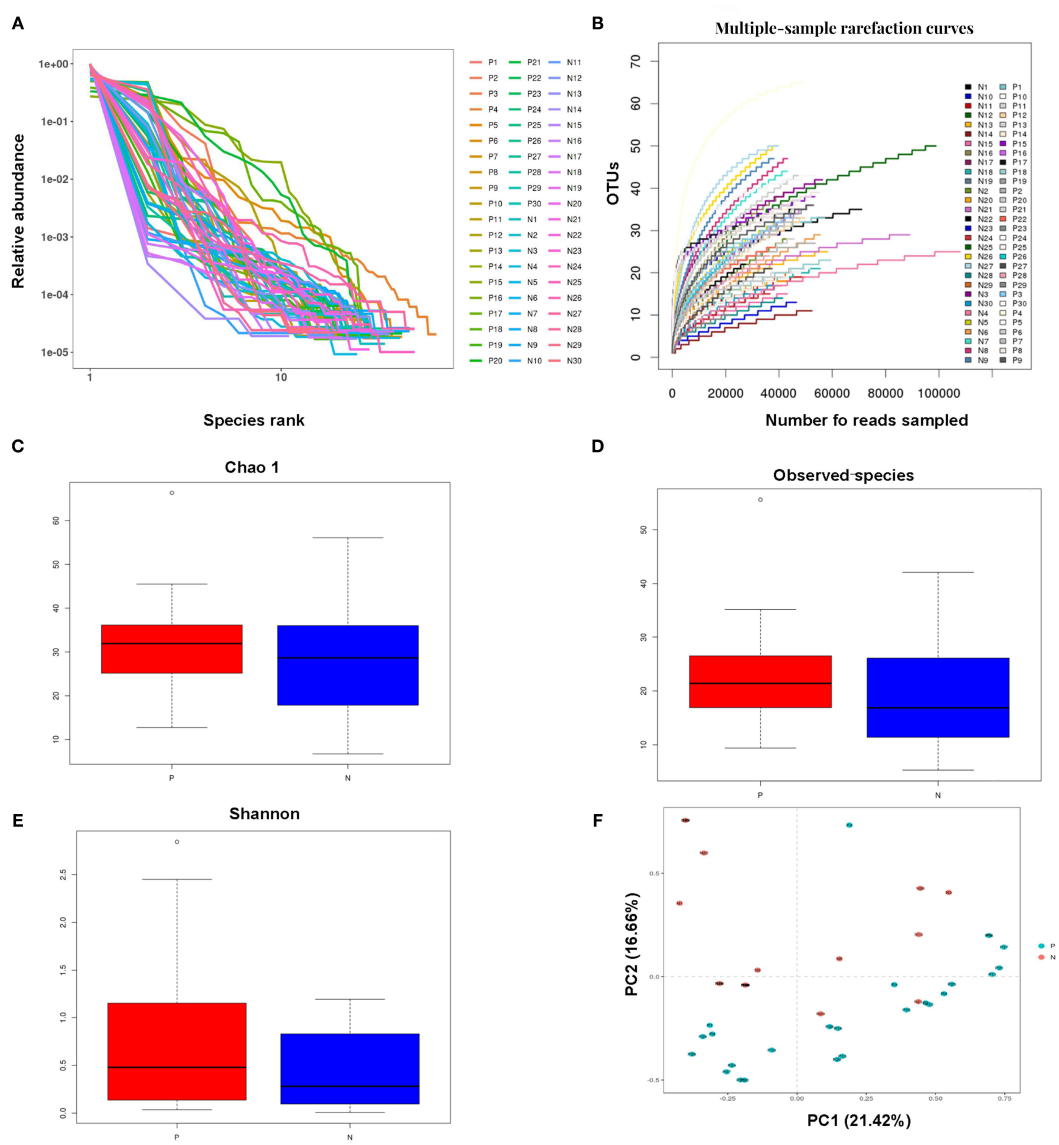
Analysis of the vaginal microbial compositions in the HPV-positive and HPV-negative groups. **(A,B)** Species accumulation curves **(A)** and rarefaction curves **(B)** for OTUs obtained from 60 cervical samples. **(C–E)** Analysis of alpha-diversity. The Chao1 **(C)** and observed species **(D)** indices were used as richness estimators, while the Shannon index **(E)** was used as a diversity estimator. **(F)** Principal coordinates analysis of the structure of the vaginal microbiota. HPV, human papilloma virus; OTU, operational taxonomic unit.

Millions of raw reads were obtained after *16S* rRNA gene sequencing. After MiSeq sequencing analysis, 2,874,102 sequences were identified from the 60 samples (average sequence length, 427.8 bp; Good coverage index, >99%). In total, 138 OTUs were identified in the two study groups. The OTU rank abundance and rarefaction curves of all the samples indicated that the data sampling was sufficient, and the sequencing depth was adequate; the *16S* rRNA gene sequences covered almost all microbial communities (Figures 1A,B).

The Chao1, observed species, and Shannon indices were all higher, but not significantly so, in the HPV-positive group than in the HPV-negative group. The alpha diversity of the vaginal microbiota did not significantly differ between the HPV-negative and HPV-positive groups. These results indicated that microbial shifts occurred in the vagina in women with HPV infection (Figures 1C–E).

Principal component analysis indicated that the vaginal microbial composition slightly differed between the HPV-negative and HPV-positive groups. Principal components 1 and 2 explained 21.42 and 16.66% of the variance between the two study groups, respectively. The impact of HPV infection on the vaginal microbial composition was generally consistent with the results of the alpha diversity analysis (Figure 1F).

### Bacterial Taxa Analysis

**Figure 2:**
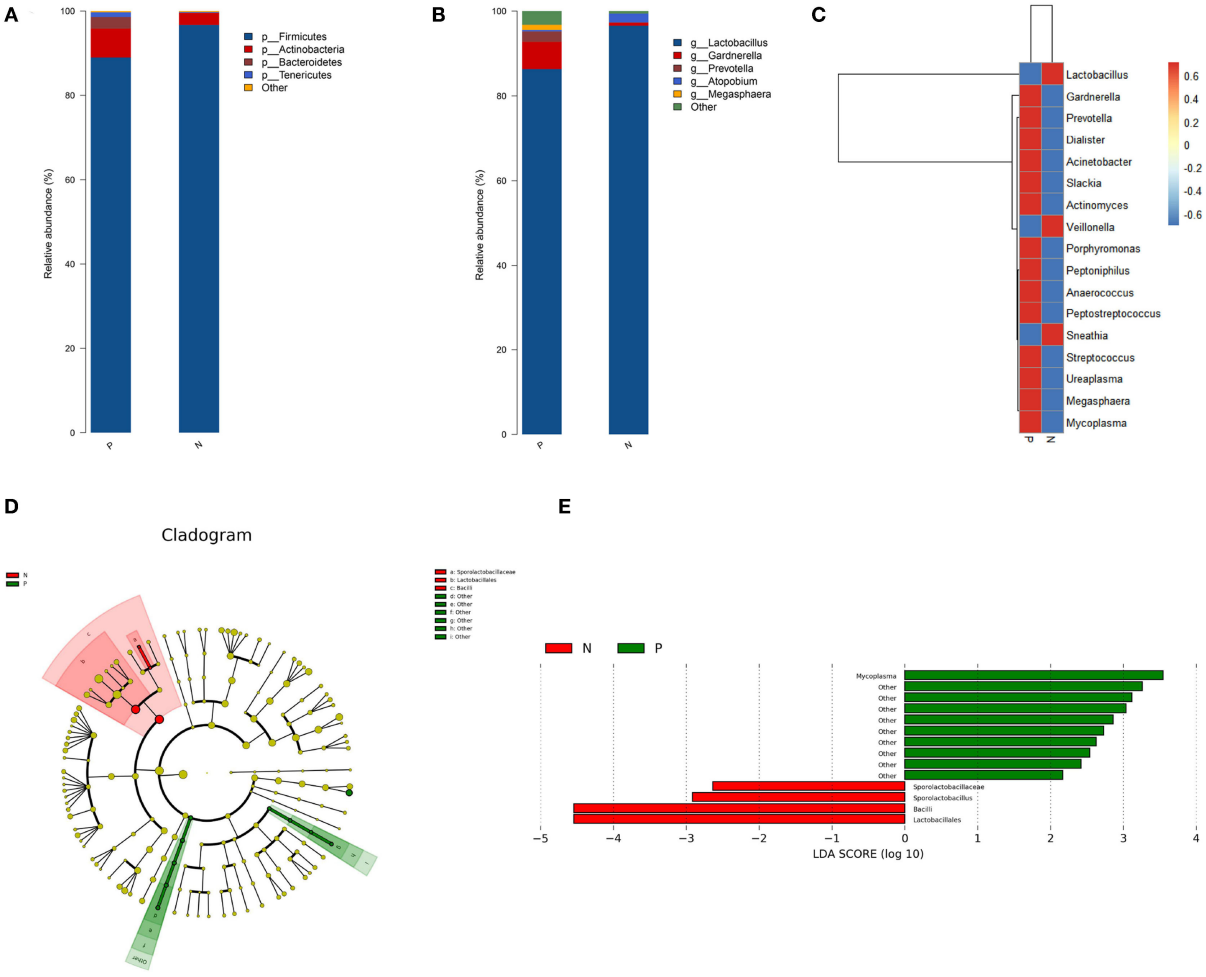
Bacterial taxa analysis of the HPV-positive and HPV-negative groups. **(A)** Relative abundances of the vaginal microbiota at the phylum level. **(B)** Relative abundances of the vaginal microbiota at the genus level. **(C)** Heatmap showing the relative abundance of each bacterial genus. **(D)** Cladogram of the LEfSe analysis of the vaginal microbiota in the two study groups. The microbial compositions were compared at different evolutionary levels. **(E)** LDA scores obtained from the LEfSe analysis of the gut microbiota in the different groups. An LDA effect size of >2 was used as a threshold for the LEfSe analysis. HPV, human papilloma virus; LDA, linear discriminant analysis; LEfSe, LDA effect size analysis.

*Firmicutes, Actinobacteria, Bacteroidetes*, and *Tenericutes* were the dominant phyla in the vaginal microbiota in both the study groups, with relative abundances of 96.71, 2.82, 0.18, and 0.06%, respectively, in the HPV-negative group, and 88.99, 6.87, 2.73, and 1.12%, respectively, in the HPV-positive group. At the genus level, *Lactobacillus* (96.57%), *Atopobium* (2.1%), *Gardnerella* (0.72%), *Prevotella* (0.04%), and *Mycoplasma* (0.0001%) dominated the microbial community in the HPV-negative group, while in the HPV-positive group, *Lactobacillus* (86.34%), *Gardnerella* (6.42%), *Prevotella* (2.44%), *Megasphaera* (1.21%), *Mycoplasma* (0.80%), and *Atopobium* (0.37%) were the dominant microflora (Figures 2A,B).

A heat map was constructed based on the abundances of the detected species and genera. The heat map revealed that in the HPV-positive group, the abundances of the following species and genera were increased: *Gardnerella, Slackia*, and *Actinomyces* belonging to *Actinobacteria*; *Prevotella* and *Porphyromonas* belonging to *Bacteroidetes*; *Dialister, Peptoniphilus, Anaerococcus, Peptostreptococcus, Streptococcus*, and *Megasphaera* belonging to *Firmicutes*; *Ureaplasma* and *Mycoplasma* belonging to *Tenericutes*; and *Acinetobacter* (*Proteobacteria*). In contrast, the abundances of *Lactobacillus* and *Veillonella* (*Firmicutes*) and *Sneathia* (*Fusobacteria*) were decreased in the HPV-positive group (Figure 2C).

The LEfSe analysis revealed that the abundance of *Mycoplasma* was significantly increased while those of Lactobacillales, Sporolactobacillaceae, and *Sporolactobacillus* were significantly decreased in the HPV-positive group as compared to the HPV-negative group (Figures 2D,E).

### Co-occurrence Network of Core Vaginal Microbiota

**Figure 3:**
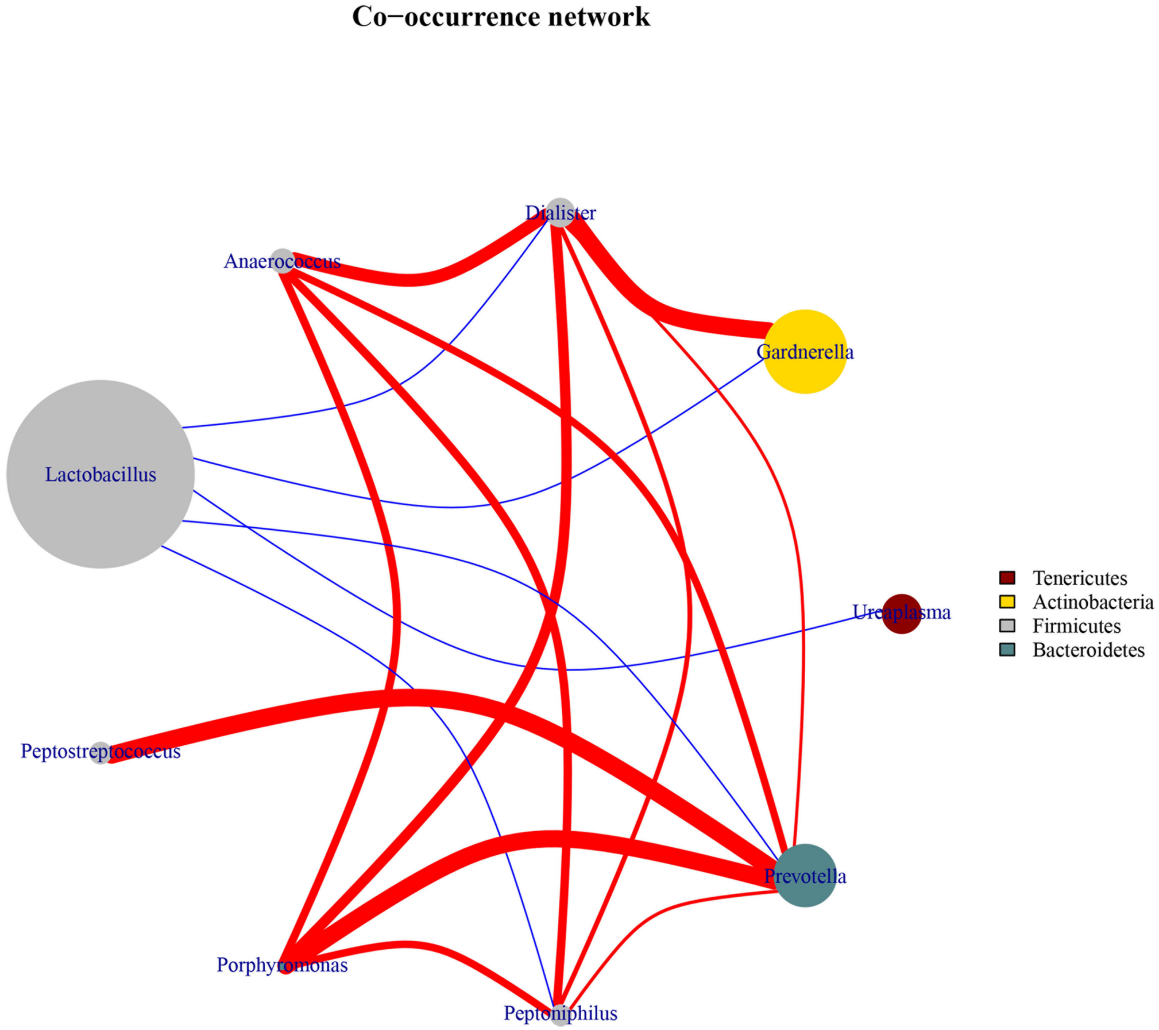
Co-occurrence network diagram of the core vaginal microbiota. The edges between the nodes represent correlations between the nodes they connect, with edge color indicating positive (red) and negative (blue) correlations, and edge shade indicating the magnitude of the correlation.

To explore the bacterial interactions of key genera in the vaginal ecosystem, we performed a bacterial community network analysis. In both the HPV-positive and HPV-negative groups, *Lactobacillus* was negatively correlated with *Dialister, Gardnerella, Ureaplasma, Prevotella*, and *Peptoniphilus*, while positive correlations were identified among the genera *Anaerococcus, Dialister, Gardnerella, Ureaplasma, Prevotella, Peptoniphilus, Porphyromonas*, and *Peptostreptococcus* (Figure 3).

### Predicted Proteins Functionally Categorized Based on COG Assignment

**Figure 4:**
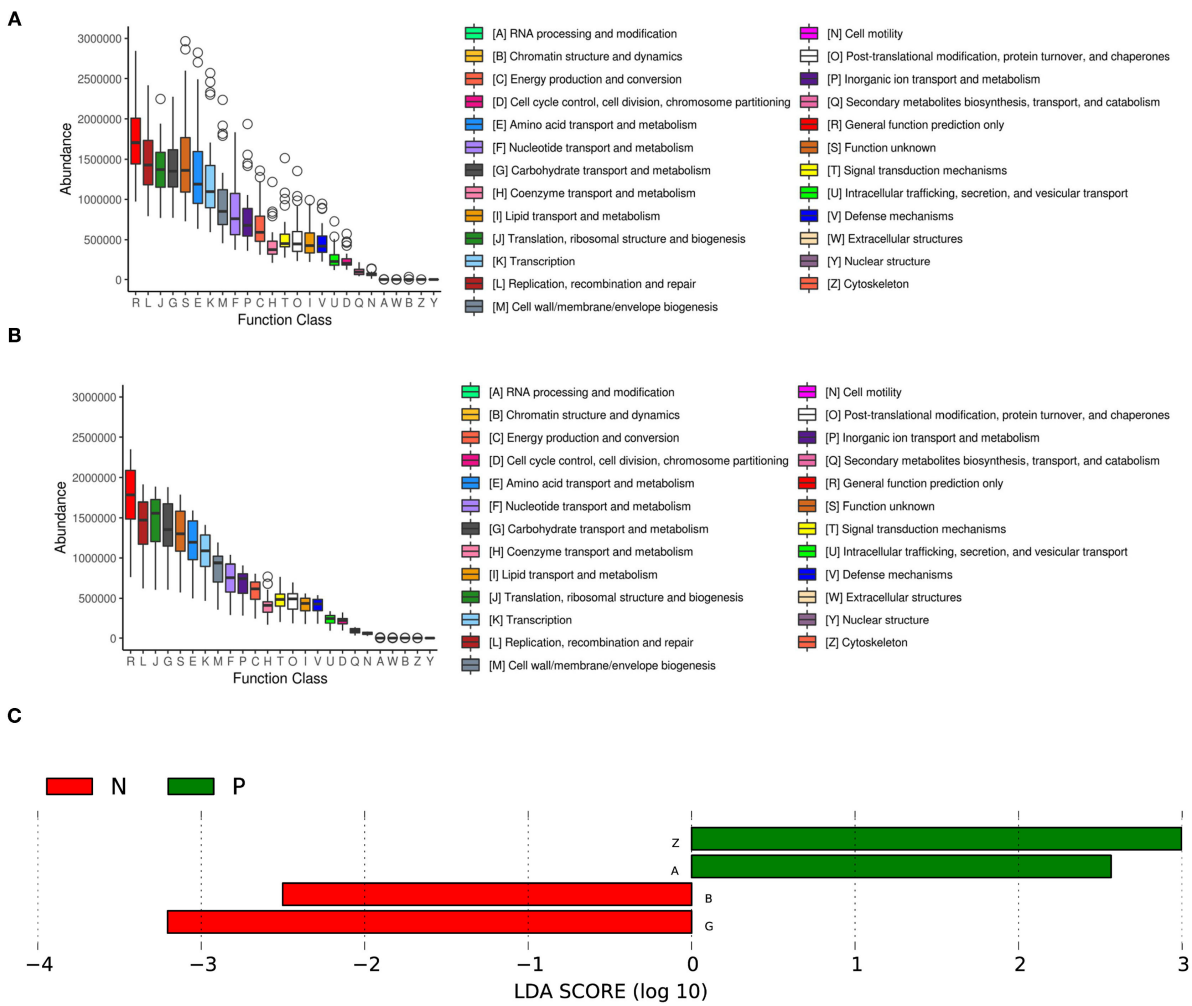
COG functional classification in the HPV-positive group **(A)** and HPV-negative group **(B)**. The boxes represent the interquartile range (IQR) between the first and third quartiles. The lines and spots inside the boxes represent the median and mean, respectively. The whiskers denote the lowest and highest values within 1.5 × IQR from the first and third quartiles, respectively. The ring above the box indicates the outliers. **(C)** LEfSe analysis of functional differences in the vaginal microbiota in the two study groups. An LDA effect size of >2 was used as a threshold for the LEfSe analysis. HPV, human papilloma virus.

We further analyzed the inferred metagenomics to identify functional differences in the vaginal microbiota between HPV-positive and HPV-negative women. In the HPV-positive group, LEfSe analysis showed that the abundances of the categories “RNA processing and modification” and “cytoskeleton” were significantly increased in the vaginal microbiota. In contrast, the categories of “Chromatin structure and dynamics” and “Carbohydrate transport and metabolism” were significantly enriched in the vaginal microbiota of the HPV-negative group (Figure 4).

## Discussion

The present study showed that perturbations in the vaginal microbiota occurred in the early stage of hr-HPV infection in the absence of CIN. High-throughput sequencing of the *16s* rRNA gene revealed that *Lactobacillus, Sporolactobacillus, Atopobium, Veillonella*, and *Sneathia* were decreased in the vaginal microbiota of HPV-positive women, as compared with HPV-negative women. In contrast, bacteria related to BV, such as *Gardnerella, Prevotella, Dialister, Slackia, Actinomyces, Porphyromonas, Peptoniphilus, Anaerococcus, Peptostreptococcus, Streptococcus, Ureaplasma, Megasphaera*, and *Mycoplasma*, were more abundant in HPV-positive women than in the HPV-negative women. These results suggest that the early phase of HPV infection is relevant to the homeostasis of the vaginal microbiota, and the predominance of some pathogenic bacteria during early HPV infection may contribute to the subsequent development of CIN lesions.

In the present study, *Firmicutes, Actinobacteria, Bacteroidetes*, and *Tenericutes* were the major phyla in the vaginal microbiota, which is in agreement with a previous study (26). *Lactobacillus*, an important member of the phylum *Firmicutes*, was the most abundant bacterial species in the vagina; this species helps maintain a low environmental pH through its metabolic activities, which inhibits the growth of pathogens and disrupts biofilms (27, 28). The obvious decline in the abundance of *Lactobacillus* in the HPV-positive group as compared to the HPV-negative group indicated that hr-HPV infection was associated with a deteriorated vaginal environment. The conspicuous decline in *Lactobacillus* was also consistent with the changes observed in the microbiota composition at the phylum level. *Atopobium vaginae*, a newly identified species belonging to the Coriobacteriaceae family, was associated with ~80% of all cases of BV in one study (29). *A. vaginae* activates the proinflammatory transcription factor NF-κB in the cervicovaginal epithelial cells, triggering abundant inflammation and innate immune responses (30–32). In a study of South African women, *Atopobium* was found to be among the most abundant taxa in cervicovaginal microbial communities associated with high levels of inflammatory mediators (33). *Sneathia sanguinegens* and *Sneathia amnii*, the only two species of *Sneathia*, secrete fibrinolysins and collagenase, which disrupt the mucosal barrier and promote vaginal epithelial cell detachment (34, 35). In addition, *S. amnii* and *S. sanguinegens* act as inflammatory modulators and upregulate interleukin (IL)-8, IL-α, IL-β, and tumor necrosis factor (TNF)-α; both species were among the 6 most prevalent proinflammatory taxa in the vaginal microbiota in one South African study (33). *Gardnerella vaginalis* is a facultative anaerobe belonging to the Bifidobacteriaceae family. *G. vaginalis* adheres to the vaginal epithelium, providing a scaffold for biofilm formation and facilitating the growth of bacteria associated with BV (36, 37). *G. vaginalis* was also among the six most prevalent proinflammatory taxa identified in the abovementioned South African study (33). *Peptoniphilus* and *Anaerococcus*, two newly identified members of the Peptoniphilaceae family, are significantly associated with BV (38). Similar to *Peptoniphilus* and *Anaerococcus, Peptostreptococcus* (Peptostreptococcaceae) is a common Gram-positive anaerobe that has not been reported to adhere to vaginal epithelial cells, but does usually adhere to epithelial cells at other body sites (39). *Mycoplasma* and *Ureaplasma* belong to the Mycoplasmataceae family. *Mycoplasma genitalium* has been associated with symptomatic urethritis and cervicitis, while *Mycoplasma hominis* has been associated with BV (40). *Ureaplasma urealyticum* induces cervicovaginal epithelial cells to produce proinflammatory cytokines and significantly increases the risk of hr-HPV infection (41, 42). The variations in genera such as *Gardnerella, Slackia*, and *Actinomyces* (*Actinobacteria*), *Prevotella* and *Porphyromonas* (*Bacteroidetes*), and *Ureaplasma* and *Mycoplasma* (*Tenericutes*) were consistent with the variations observed in the microbiota composition at the phylum level.

LEfSe analysis indicated that *Mycoplasma* (Mycoplasmataceae), Lactobacillales, Sporolactobacillaceae, and *Sporolactobacillus* (Sporolactobacillaceae) were candidate biomarkers for HPV infection. The rate of *Mycoplasma* infection is higher among women with high-grade squamous intraepithelial lesions (HSILs) than among women with low-grade squamous intraepithelial lesions (LSILs); however, in patients with cervical cancer, no *Mycoplasma* (*M. hominis* or *M. genitalium*) infections were detected (43). Thus, it can be inferred that *Mycoplasma* plays a major role in the early phase of HPV infection, rather than affecting the development of cervical carcinoma. A significant increase in *Mycoplasma* abundance may facilitate virus penetration, survival, and persistence (42, 43). *Sporolactobacillus* resembles *Lactobacillus* in appearance and metabolism, except that it produces endospores and is aerobic (44). Spore-forming *Sporolactobacillus* has been extensively studied and has even been commercialized as a probiotic. It has also been modified as a surface expression vector to deliver immunogenic HPV compositions to prepare an HPV vaccine (45). Significant reduction in *Sporolactobacillus* abundance may indicate a worse vaginal environment that promotes the growth of pathogenic bacteria (46).

The complex interactions between the vaginal microbiota, HPV, CIN, and cancer have recently been investigated. Mitra et al. (47) reported that the progression of CIN lesions was correlated with increased diversity of the vaginal microbiota and decreased abundance of *Lactobacillus* spp.; the authors also identified *S. sanguinegens, Anaerococcus tetradius*, and *Peptostreptococcus anaerobius* as biomarkers of HSILs. In the present study, the genera *Anaerococcus* and *Peptostreptococcus* showed the same increasing trends in the early phase of HPV infection; however, the relative abundance of *Sneathia* decreased in the HPV-positive group. Oh et al. (48) reported that the increased abundance of *G. vaginalis, A. vaginae*, and *L. iners* in the cervical microbiota along with the reduced abundance of *L. crispatus* was linked to a risk of CIN, which indicates that bacterial dysbiosis in the presence of hr-HPV infection might be a risk factor for cervical neoplasia. *G. vaginalis* showed similar results in our research, while *A. vaginae* showed a contradictory result: in the early phase of HPV infection, the abundances of the proinflammatory bacteria *Sneathia* and *Atopobium* decreased. It is difficult to elucidate the dynamics and mechanisms of these two genera in disease. As the COG category “RNA processing and modification” was significantly enriched in the HPV-positive group, we speculated that in the initial period of hr-HPV infection, the virus invaded epithelial cells and induced a proinflammatory environment, which facilitated the integration of viral DNA into the host cells (49, 50) and caused excessive burden for the host. This led to a robust host immune response, which probably resulted in the observed decrease in the abundances of the proinflammatory genera *Sneathia* and *Atopobium*.

Our study focused on analyzing the differences in the vaginal microbiota between HPV-negative women and HPV-positive women with normal cytology. We found that hr-HPV infection was associated with vaginal bacterial dysbiosis. Certain bacteria associated with BV showed increased abundance, while *Lactobacillus* spp. showed decreased in the early phase of HPV infection, leading to alterations in the vaginal microbiome. We did not investigate the interplay between the vaginal microbiota, hr-HPV infection, CIN lesions, and cervical cancer. In the future, we plan to identify causal connections among HPV infection, CIN status (1+, 2+, 3+), cervical cancer, and the vaginal microbiota.

## Conclusions

The present study demonstrated that vaginal microbial perturbations occurred in the early phase of hr-HPV infection when no CIN lesions had developed. The abundance of probiotics such as *Lactobacillus* and *Sporolactobacillus* decreased, while the abundance of BV-related pathogenic bacterial genera increased. By studying the early effects of hr-HPV infection on the vaginal microbiota, we provide candidate microbial markers to monitor persistent HPV infection and the development of cervical neoplasms.

## Data Availability Statement

The datasets generated for this study can be found in online repositories. The names of the repository/repositories and accession number(s) can be found below: (NCBI Sequence Read Archive database and SRA accession number-SRP212297, BioProject accession number-PRJNA551647).

## Ethics Statement

The studies involving human participants were reviewed and approved by the ethics committee of the First Hospital of Jilin University. The patients/participants provided their written informed consent to participate in this study.

## Author Contributions

All persons who meet authorship criteria are listed as authors, and all authors certify that they have participated sufficiently in the work to take public responsibility for the content, including participation in the concept, design, analysis, writing, or revision of the manuscript. Furthermore, each author certifies that this material or similar material has not been and will not be submitted to or published in any other publication before its appearance in the Frontiers in public health.

## Conflict of Interest

The authors declare that the research was conducted in the absence of any commercial or financial relationships that could be construed as a potential conflict of interest.
